# The Skin and Oral Hydration Benefits of Caryophyllene Through Aquaporins 3 and 5 Type Modulation in Keratinocytes: *in silico* and in Vitro Research

**DOI:** 10.1111/jocd.70955

**Published:** 2026-06-05

**Authors:** Alina Yarovaya, Elizaveta Patronova, Egor Ilin, Viktor Filatov, Barbara de Freitas Carli, Samara Eberlin

**Affiliations:** ^1^ Science Center SkyLab AG Lausanne Switzerland; ^2^ Department of Research Kosmoscience Group Campinas Brazil

**Keywords:** aquaporins, caryophyllene, claim substantiation, computer modeling, hydration, skin physiology

## Abstract

**Objective:**

Skin xerosis and xerostomia are partially associated with decreased expression and molecular activity of aquaporins 3 and 5 types (AQP3 and AQP5) in skin epidermal cells. While cosmetic occlusive agents and emollients are effective in improving symptoms of skin dryness, maintaining intensive water transport and physiological mechanisms of hydration is also crucial for long‐term skin health. This study aimed to investigate novel skin benefits of caryophyllene (CP) from *Eugenia caryophyllata* leaves by regulation of AQP3 and AQP5 in human keratinocytes and its preliminary dermatological safety.

**Methods:**

Various experimental techniques, including MTT assay (cell viability) and ELISA analysis, were used to assess the molecular mechanisms of skin hydration effects of CP individually and in the cosmeceutical formulation on human keratinocytes. The quantitative and qualitative analysis of CP was performed using gas chromatography–mass spectrometry (GC–MS). Affinity scores and targeted action of CP were predicted using a complex of molecular modeling of DiffDock with GNINA, molecular dynamics, and MM/PBSA analysis. To evaluate dermatological tolerance and allergenic potential, the clinical semi‐occlusive patch test of the cosmeceutical formulation containing CP was used.

**Results:**

According to the cell viability assay, CP showed a good cytotoxicity profile in a concentration range from 0.001 to 0.03 mg/mL on HaCaT keratinocytes, despite being a concentrated substance with polypharmacological activities on skin. The CP in a concentration of 0.01 mg/mL promoted an increase of AQP3 and AQP5 in skin keratinocytes by 99.97% and 332.26%, respectively, compared to basal control (*p* < 0.01). Its effect was comparable to that of the *Aloe barbadensis* leaf extract at 2.5 mg/mL on AQP5, with an increase of 345.24% (*p* < 0.01). Moreover, the cosmeceutical formulation with CP in a concentration of 0.1 mg/mL was able to increase AQP3 amount by 100.15% in HaCaT keratinocytes, confirming promising skin hydration potential. It was clinically confirmed that the formulations with CP revealed good dermatological tolerance without negative skin reactions on the group of healthy volunteers.

**Conclusion:**

CP exerts promising hydration benefits through AQP3 and AQP5 modulation in keratinocytes in vitro and could be safely used in cosmetic formulations. These findings support application as a novel phytochemical for skin and oral conditions, while underscoring the need for further clinical investigation into dermal efficiency and penetration, optimized dosing strategies, and broader biochemical pathways to consider all aspects of use.

## 1 Introduction

Aquaporins (AQPs) are a family of transmembrane proteins that are responsible for cross‐membrane transport of water and some small molecules [1]. AQPs are found in all groups of living organisms such as microorganisms, fungi, plants, and animals including humans [2]. These proteins are characterized by a highly conserved specific tetrameric organization, where each of the four subunits represents a separate pore, and the fifth pore is located in the center of the tetramer [3]. Depending on the structure, different types of AQPs are distinguished, and 13 such isoforms (AQP0–AQP12) are known for mammals [4]. AQPs of different types are expressed throughout the entire body, including the nervous system, kidneys, gastrointestinal tract, male and female reproductive organs, etc. [1]. One of the most abundant AQPs in the skin and salivary glands, respectively, are AQP3 and AQP5 [5, 6].

The skin is the largest organ of the human body, which most often faces the negative effects of the external environment; therefore, optimal hydration is necessary to maintain its structure [7]. AQP3 can be expressed by keratinocytes of the basal layer (both on the surface and inside the cells) and keratinocytes of the stratum spinosum (only on the surface of the cell membrane) [5]. To date, only a few mechanisms of AQP3 expression regulation are known, e.g., histone deacetylase inhibition promotes AQP3 overexpression, as well as p53 transcription factors [8]. However, it is well known that AQP3 plays a crucial role in maintaining the viability of skin cells, including the survival of skin carcinoma cells depends on the abundance of its expression [9]. AQP3 upregulation is associated with the severity of dermatological diseases such as rosacea, atopic eczema and psoriasis [10, 11, 12], while its downregulation is associated with vitiligo and accelerated skin aging [12, 13]. Since AQP3 is also glycerol transporter, its moisturizing function impairment may lead to another skin pathology—xerosis cutis [14].

The water homeostasis maintenance is no less important in mucosal tissue, which is particularly at risk of dehydration due to its structure [15]. AQP5 is mainly located on the apical membranes of acinar cells of secretory glands [16]. One of the main factors reducing AQP5 expression is bacterial lipopolysaccharides of oral pathogens, which induce the inflammatory process and, through MAPK and NF‐κB pathways, lead to downregulation of AQP5 transcription [17]. AQP5 deficiency could be involved in the exacerbation of xerostomia and Sjogren's syndrome [18, 19].

Thus, AQP3 and AQP5 dysfunction is associated with the progression of skin and mucosal pathologies, which, in addition to physical discomfort, can have a negative effect on a consumer's and patient's social life [20, 21]. According to established dermatological routine, the main therapeutic approach for skin xerosis is use of emollients, but recent data show that the clinical results of their application are mixed. In addition, some emollients cannot affect the source of pathogenesis if it is at the cellular level [22]. In this regard, a new and relevant direction is the development of AQP‐regulating substances for daily care.

To the best of our knowledge, there are not currently available AQP‐targeting drugs, although some of the FDA‐approved drug molecules may influence AQP5 [23]. Recently, BASF SE introduced a cosmetic ingredient based on glyceryl glucoside named Hydagen Aquaporin, but its activity against AQP3 was found to be limited [24].

Various molecules and active ingredients of natural origin are now being actively studied for their effects against AQPs. For example, Chimpi (dried citrus peel) and asiaticoside from *Centella asiatica* have demonstrated activating effects on AQP3 in vitro [25, 26]. In contrast, rottlerin polyphenol from *Mallotus philippinensis* has an inhibitory effect on AQP3 [27]. It was also demonstrated that epigallocatechin gallate downregulates AQP5 expression, which could be useful for tumors and Sjogren's syndrome therapy [28, 29].

Another promising but still poorly studied plant in terms of AQPs‐modulating properties is clove ( *Eugenia caryophyllata* or *Syzygium aromaticum* ) widely known for its medicinal and cosmetic properties. Clove essential oil contains plenty of phenylpropanoids (carvacrol, thymol, eugenol and cinnamaldehyde) [30] and terpenes (β‐caryophyllene, β‐caryophyllene oxides, α‐humulene, eugenol) [31]. Numerous studies have shown these substances to have antioxidant [32, 33], antifungal, antibacterial [34, 35], anti‐inflammatory [36], analgesic [37] and anticarcinogenic [31, 38] effects useful for skin care.

One of the valuable phytoconstituents contained in clove essential oil is caryophyllene (CP) [39]. In addition to clove, this bicyclic sesquiterpene is found in many medical plants including *Cinnamomum* spp., *Ocimum* spp., *Piper* spp., *Coriandrum sativum* L., *Lavandula angustifolia, Origanum vulgare* L. [40, 41]. It has long been known that beta‐caryophyllene is a selective agonist of cannabinoid type 2 receptors (CB2R) [40], involved in the regulation of multiple biochemical pathways. In addition, beta‐caryophyllene as a single substance has immunomodulatory, neuroprotective, anti‐inflammatory, gastroprotective, anxiolytic, antibacterial, antiviral, and oncotoxic properties [41, 42, 43].

Therefore, the aim of this study was to investigate the targeting biological activity of CP on AQP3 and AQP5 in keratinocytes in vitro and evaluate its safety as an active substance of pharmaceutical and cosmetic formulations for skin hydration.

## 2 Materials and Methods

### 2.1 Chemicals

All chemicals of analytical grade used in the experiments were purchased from Sigma‐Aldrich (Sigma Chemical Co. Ltd., St. Louis, MO, USA). Caryophyllene extracted from *Eugenia caryophyllata thunb*. leaves essential oil (CAS 87–44‐5) and standardized for total amount of α‐caryophyllene and β‐caryophyllene exceeding 90% was provided in terms of collaborative research by Ventos (Ernesto Ventos S.A., Barcelona, Spain), hereinafter referred to as CP. *Aloe vera* leaf extract (CAS 85507–69‐3 or 94 349–62‐9) with addition of water and glycerin and trimethylglycine (CAS 107–43‐7) was obtained from Bell Flavors & Fragrances GmbH (Leipzig, Germany) and IFF's Health & Biosciences (Naantali, Finland). Glyceryl glucoside named Hydagen Aquaporin used in the study as active control was purchased from BASF (BASF SE, Germany).

### 2.2 GC–MS Analysis of CP

The gas chromatography–mass spectrometry (GC–MS) analysis of the sample of CP from *Eugenia caryophyllata thunb*. leaves was performed using the gas chromatograph mass spectrometer GCMS TQ 8040 system (Shimadzu, Kyoto, Japan) coupled with the mass spectrometer QP‐2010 Ultra (Shimadzu, Japan). The polar capillary column Rtx‐5 Amine (30 m x 0.25 mm i.d., the film thickness of 0.5 μm) was used. The carrier gas was helium of 99.99% purity with a flow rate of 1.2 mL/min. Analysis was performed under the following conditions: split ratio—1:100, temperature program—from 30°C (1 min) to 300°C (10 min) at a rate of 10°C/min. Mass spectra were recorded in the electron impact mode at 70 eV; scanned mass range was 35–500 Da. The full chemical composition of phytochemical was determined and compared using mass spectra NIST 2017 and Wiley‐08 databases [44, 45].

### 2.3 Molecular Docking Studies

**FIGURE 1:**
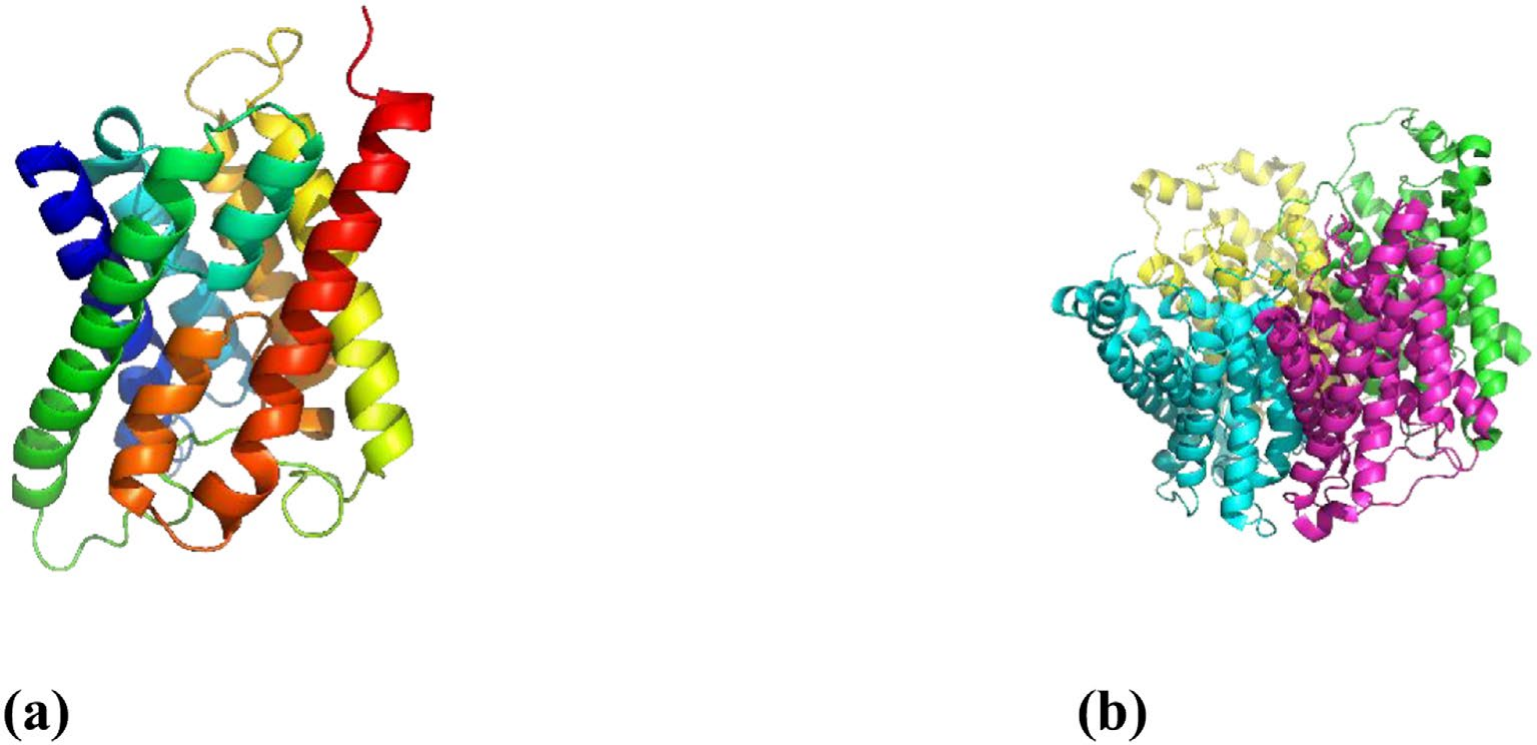
3D structure from Protein Data Bank: (a) AQP3; (b) AQP5.

The three‐dimensional structures of the AQP3 protein (PDB ID: 3LLQ) (Figure 1a) and the AQP5 protein (PDB ID: 3D9S) (Figure 1b) were obtained from the Protein Data Bank (PDB) for structural fitting and subsequent docking analyses [44, 45]. Protein structure was acquired in the standard .pdb format. The preparation process involved the removal of water molecules, excision of superfluous chains, and the addition of polar hydrogens and charges. The refined protein structures were then saved in .pdb for further *in silico* studies. Molecular docking experiments were conducted using a personal computer equipped with an Intel Core i7‐12700U CPU (2.3 GHz) and 16 GB of RAM, running Windows 11 (64‐bit OS). To validate the docking procedure, the native protein ligand was initially docked to ensure methodological consistency.

Subsequent molecular docking of AQP3 and AQP5 was performed with four ligands: trimethylglycine, glyceryl glucoside, aloin from *Aloe barbadensis* plant, and (−)‐caryophyllene. A comprehensive computational methodology was implemented in this investigation for the execution of molecular docking analyses. A multi‐tiered protocol was employed, wherein the DiffDock model [46] (4.2 version) was selected due to its optimal suitability for blind docking methodologies, in which no a priori knowledge regarding binding site localization is presumed. This diffusion‐based algorithmic approach was utilized for the prediction and characterization of ligand positioning within protein binding cavities. In the docking process, the DiffDock algorithm created ten conformations and calculated the DiffDock‐confidence score for each of them. Subsequently, quantitative assessment of protein‐ligand binding affinities was performed utilizing GNINA [47], a scoring function operating on deep learning principles and calculated specifically for the top‐rank (higher DiffDock‐confidence score) conformation derived from DiffDock.

Concurrently, systematic analysis of protein structure surface interactions was conducted through the application of MASIF (Molecular Surface Interaction Fingerprinting) [48].

### 2.4 Performing Molecular Dynamics and MM/PBSA Analysis

The highest‐scoring docking poses of (−)‐CP were further refined through molecular dynamics (MD) simulations using GROMACS v.2023.1 [49] with MPI support. Each protein–ligand complex was set up for MD simulation employing the CHARMM36 force field [50] for the protein and CGenFF parameters [51] for the ligand. The systems were embedded in a cubic solvent box filled with TIP3P water molecules [52], ensuring a minimum padding of 1.0 nm between the protein surface and the box edges. System neutrality was achieved by introducing counterions.

Initial geometry optimization was carried out via the steepest descent algorithm, followed by equilibration in the NVT ensemble (100 ps) and then in the NPT ensemble (100 ps). A production MD trajectory was generated for 20 ns under NPT conditions at 298.15 K and 1 bar, with temperature and pressure regulated by the Nosé–Hoover thermostat [53] and the Parrinello–Rahman barostat [54], respectively. Coordinates were recorded every 10 ps for downstream analyses.

Binding free energies were estimated using the MM/PBSA approach [55] as implemented in gmx_MMPBSA v.1.6.4 [56]. The calculation was based on the final 10 ns of each production trajectory, sampling frames at 100‐ps intervals. The Poisson–Boltzmann (PB) implicit solvation model was used with a grid spacing of 0.5 Å and an ionic strength of 0.1 M [57].

### 2.5 Keratinocytes Cell Culture and Growth Conditions

HaCaT human keratinocytes purchased from MatTek (MatTek Europe, Bratislava, Slovak Republic) were grown in 75 cm^2^ containers (Corning, USA), cultivated and expanded in an incubator at 37°C in the presence of 5% CO_2_, using a Dulbecco's modified Eagle's medium (DMEM) containing 4.5 g/L glucose (Thermo Fisher Scientific Inc., Waltham, MA, USA), supplemented with 10% fetal bovine serum (FBS; Thermo Fisher) and 1% penicillin/streptomycin/amphotericin (Lonza Walkerville Inc., Salisbury, MD, USA). When confluence was reached, the cells were seeded in 96‐well plates (Corning, USA) to determine the non‐cytotoxic concentrations of the evaluated product and in 24‐well plates (Corning, USA).

### 2.6 Cytotoxicity and Cell Viability Assay

Cell viability of HaCaT keratinocytes was determined colorimetrically using MTT (3‐[4,5‐dimethylthiazol‐2‐yl]‐2,5 diphenyl tetrazolium bromide) dye (Sigma Chemical, St. Louis, Mo., USA). The preparation was treated for the test in dimethylsulfoxide culture medium and added to a 96‐well plate at serial dilutions ranging from 1.000 to 0.0032 mg/mL using a dilution factor of 3.16. Pre‐culture was carried out for 48 h before the test. Then MTT was added to the culture at a concentration of 0.75 mg/mL (100 μL/well) and incubated for another 4 h. The contents of the well were removed, and 100 μL of isopropanol was added to solubilize the formed formazan crystals. Absorbance was determined in each well at 570 nm using a Multiskan GO monochromator (Thermo Scientific, Finland). Cell viability was expressed as a percentage relative to the basal control. Three biological replicates were made for further statistical analysis.

### 2.7 Quantification of AQP3 and AQP5 in Skin Keratinocytes Treated With CP

To determine the amount of AQP3 and AQP5, HaCaT keratinocytes were cultivated under standard conditions: humidified atmosphere with 5% CO_2_ at 37°C. HaCaT cells were added to a 96‐well plate at a density of 10^5^ cells per well. The next day, the cell medium was removed and replaced with 50 μL of fresh cell medium DMEM + 5% FBS to maintain cell growth. A standard 48‐h incubation was carried out after cells treatment with 50 μL of the following test compounds: CP—0.001, 0.0032 and 0.010 mg/mL; *Aloe vera* leaf extract—0.25 and 10.00 mg/mL, glyceryl glucoside—10.0 mg/mL, trimethylglycine—10 mg/mL, *Aloe vera* leaf extract and trimethylglycine in a ratio of 1:1–0.25 mg/mL. After this period, the lysate from cell cultures was collected to quantify the AQPs in skin keratinocytes by ELISA (enzyme‐linked immunosorbent assay) method, using commercially purchased kits for AQP3 (Elabsience, USA) and AQP5 (FIneTest, China). Absorbance reading was performed on a Multiskan GO monochromator (Thermo Scientific, Finland). Three biological replicates were made for further statistical analysis.

### 2.8 Topical Formulations for the GC–MS Analysis, in Vitro and Dermatological Tolerance Test

Cosmeceutical formulations containing CP for the semi‐occlusive patch test were used as shown in Table 1. The shower gels have a specific herbal smell due to CP in the composition. The prepared formulations contained basic ingredients, such as anionic, amphoteric, and nonionic surfactants of natural origin according to ISO 16128 standard. Additionally, humectant, solubilizing agent, conditioning agent, preservatives, chelating agent, antioxidant, and pH regulator were used for the final formulations.

**TABLE 1:** Shower gel formulations with CP tested in vitro and in the semi‐occlusive patch test.

Ingredient and its form	Function	Formulation, wt.%	
		Shower gel for sensitive skin	Shower gel for dry skin
Purified water	Diluent	Up to 100	Up to 100
Sodium coco‐sulfate (100% granulated powder)	Anionic surfactant	5.70	5.70
Decyl glucoside (50%–55% water solution)	Nonionic surfactant	5.00	5.00
Cocamidopropyl betaine (40%–45% water solution)	Amphoteric surfactant	8.50	8.00
Glutamate diacetate tetrasodium Salt (47% water solution)	Chelating agent	0.20	0.10
Caryophyllene from *Eugenia caryophyllata* (liquid)	Active substance for modulation of AQP3	0.03	0.10
Citric acid monohydrate (powder)	pH regulator	0.4–0.6	0.4–0.6
Potassium sorbate and sodium benzoate (water solution)	Preservative	0.70	0.70

### 2.9 GC–MS Analysis of CP in Cosmeceutical Formulations

The GC–MS analysis of CP in concentrations of 0.05 wt.% and 0.1 wt.% in the cosmeceutical formulations was performed using the gas chromatograph mass spectrometer GCMS TQ 8040 system (Shimadzu, Kyoto, Japan) coupled with the mass spectrometer QP‐2010 Ultra (Shimadzu, Japan). Both samples were analyzed without additional dilution. The polar capillary column Rtx‐5 Amine (30 m x 0.25 mm, the film thickness of 0.5 μm) was used. The carrier gas was helium with a flow rate of 1.2 mL/min. Analysis was performed under the following conditions: split ratio – 1:100, temperature program—from 30°C (1 min) to 300°C (10 min) at a rate of 10°C/min. Mass spectra were recorded in the electron impact mode at 70 eV; scanned mass range was 35–500 Da. The chemical composition was determined using mass spectra NIST 2017 and Wiley‐08 databases [44, 45]. The determination of components in the sample was carried out by the method of standard additions with 40.7 mg CP additive and 519 mg sample. The detection limit for CP oxides was 6.5 mg/kg by this analytical technique.

### 2.10 Quantification of AQP3 in Skin Keratinocytes Treated With the Formulation With CP

To determine the amount of AQP3 after single exposure of the shower gel formulation with CP for dry skin and evaluate the moisturizing properties of (Table 1), HaCaT keratinocytes were grown in 75 cm^2^ containers (Corning, USA), cultivated and expanded in an incubator at 37°C in the presence of 5% CO_2_, using a specific culture medium. When confluence was reached, the cells were seeded in 96‐well plates (Corning, USA) to determine the non‐cytotoxic concentrations of the evaluated product and in 24‐well plates (Corning, USA). Human keratinocyte cell cultures were incubated with 3 non‐cytotoxic concentrations of the evaluated samples determined by the MTT assay. The concentrations of the evaluated product were 0.10, 0.032 and 0.01 mg/mL. The cells were kept in contact with the tested substance for 48 h to evaluate long‐term exposure consequences and cell morphology changes in first approximation. After this period, the lysate from cell cultures was collected to quantify the proposed mediator as AQP3. The production of AQP3 was measured using a sandwich ELISA assay, using a commercially purchased kit (Elabsience, USA). Absorbance reading was performed on a Multiskan GO monochromator (Thermo Scientific, Finland). Three biological replicates were made for further statistical analysis.

### 2.11 Semi‐Occlusive Patch Test of Cosmeceutical Formulations With CP

For dermatological tolerance assessment, 0.03 wt.% or 0.10 wt.% CP was added to the shower gels formulated as previously described (Table 1) [24]. The irritation potential of two skin care formulations containing 0.03 wt.% or 0.10 wt.% CP was assessed using a semi‐occlusive epicutaneous assay. The test was carried out in compliance with the most recent recommendations of the Helsinki Declaration and has followed the “Guidelines for the Assessment of Skin Tolerance of Potentially Irritant Cosmetic Ingredients”, COLIPA, 1997 [58]. In particular, to comply with ethical requirements imposed on human studies, the following criteria were applied: all subjects were informed about the purpose and type of study including possible risks and all freely gave their informed consent; before the subjects were exposed to the product, information on the toxicological profile of the product was provided; all necessary precautions were taken to avoid excessive skin reactions or adverse effects on the health of subjects during the study; security measures were prepared for the case of potential adverse reactions.

Twelve healthy people of both genders, without allergological history, aged 18 to 55 years, were selected for the study. The following criteria of exclusion were applied: pregnant women or women who are nursing; subjects with blemishes or marks including tattoos, scars or sunburn on the test site(s) since this can interfere with scoring; medication (local and/or systemic) which may affect skin response; signs of irritated skin at the test sites; any active skin disease which may interfere with the aims of the study; any participation in simultaneous studies that might interfere with test evaluation or participation in a previous study without an appropriate period of rest since.

Test products were applied to the skin of the outer arm of each subject using adhesive patch test strips (Micropore, 3 M) in sufficient amounts to fill one test disc (approximately 0.2 g or 0.2 mL). The skin at the application spot (back/inner forearm/outer arm) was healthy and without lesions. Subjects were advised to use caution in handling the applied contact tests. During tests, subjects avoided contact with water and intense physical exercise. The products were kept in contact with the skin for 48 h under a semi‐occlusive patch. Observation of the effects caused by the application of the test substances was performed 15 min, 1 h and 24 h after the patch removal. Evaluating skin reactions, the dermatologist assessed all signs of the irritating and sensitizing effects of the tested products, i.e., erythema, desquamation, oedema, vesicles, etc.

### 2.12 Statistical Analysis

All in vitro study experimental data were presented as figures of bar plots using mean value ± standard deviation (SD) of at least three independent experiments (GraphPad Prism version 8.0.1 for Windows, GraphPad Software, Boston, Massachusetts USA). Statistical evaluation ANOVA test processed using the Statistica software package (StatSoft, USA, Ver. 8.0) was used allowing the measurement of variation of the results, comparing the data between all the groups. The Bonferroni post‐test was then applied, which reinforced and made even more accurate the result presented in the ANOVA test. For both analyses, the significance level of 5% was used. ELISA data normality was controlled with the Shapiro–Wilk test.

In the in vivo test, the statistical calculation was straightforward—a summative aggregate index combining mean erythema and mean oedema scores. The study employed descriptive statistics only, on the categorical outcome according to COLIPA guidelines. The traditional hypothesis testing was not applicable.

## 3 Results and Discussion

### 3.1 GC–MS Analysis of CP

The chemical composition of CP extracted from *Eugenia caryophyllata thumb*. leaves was analyzed using GC–MS (Table 2). The components and detailed composition of CP were identified and quantified using retention time (RT, min) and peak area values, respectively. Under GC–MS experimental conditions, 6 main constituents were identified in the tested CP sample. Total CP including beta‐caryophyllene (BCP) and alpha‐caryophyllene (ACP) had a content estimated at 93.219%. Alpha‐humulene named ACP was detected in the amount of 32.702%, whereas BCP was detected in the amount of 60.517%. Other trace ingredients, such as alpha‐cubebene, alpha‐copaene, and total sum of CP oxide isomers, did not exceed 6.202% of total amount. CP oxide isomers are the main oxidizable compounds during long‐term storage which could cause allergic contact dermatitis, so their low content (< 1%) is an advantage of the investigated plant‐derived substance [59] for further standardization and quality control.

**TABLE 2:** The constituents of BCP sample identified by GC–MS.

No	Compound	RT, min	*m/z*	Content, g/kg	Relative content, %
1	α‐Cubebene	16.169	105.00	7.16	0.716
2	α‐Copaene	16.638	105.00	46.01	4.601
3	trans‐Caryophyllene	17.144	93.00	5.78	0.578
4	(−)‐Caryophyllene (CP)	17.408	133.00	605.17	60.517
5	α‐Humulene (α‐caryophyllene, ACP)	17.846	93.00	327.02	32.702
6	(−)‐Caryophyllene oxide isomers	19.213 19.603 19.657	79.00	8.86	0.886

### 3.2 Molecular Docking/Molecular Dynamics of BCP Interaction With AQP3 and AQP5

**FIGURE 2:**
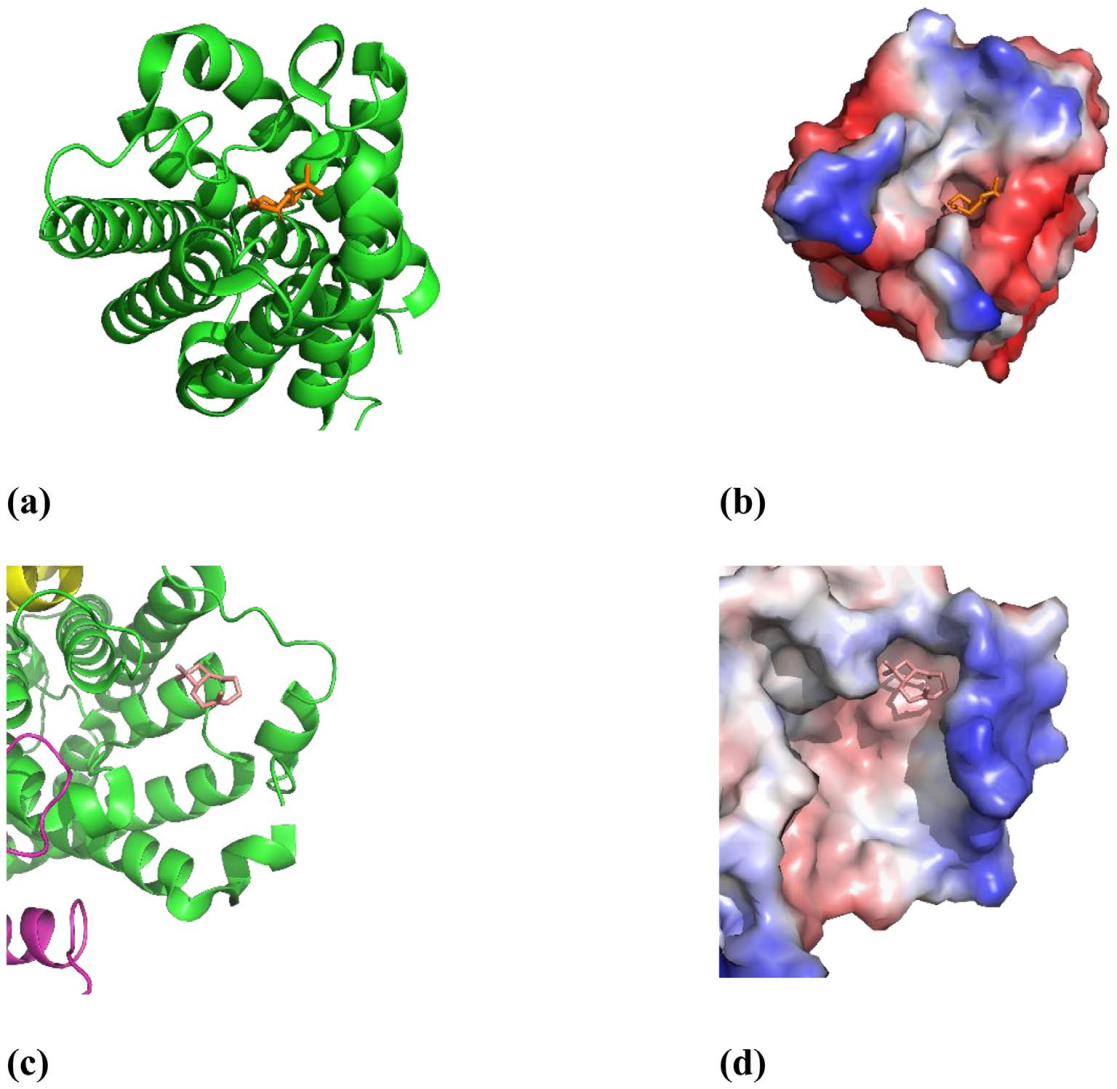
Docking studies of CP: (a) visualization with AQP3 loops; (b) interaction with AQP3 cavity; (c) visualization with AQP5; (d) interaction with AQP5 cavity. Red color shows active site, blue color shows nonactive zone of AQPs.

Molecular docking is an effective tool to predict molecular interactions for both drug discovery and rational design before in vitro and in vivo studies. Considering the significance of AQPs in maintaining normal skin and mucous membrane physiology, molecular docking analysis was performed to verify CP could interact with AQP3 and AQP5 structures. The crystal structure of AQP3 and AQP5 with pdb was used for analysis. A comparative study using CP, aloin from *Aloe barbadensis* plant, trimethylglycine and glyceryl glucoside served as control was performed in DiffDock software because of confirmed activity on AQPs [24, 60]. Docking of AQP3 and AQP5 with selected ligands revealed that all compounds interact with target proteins with affinity scores ranging from −2.7 to −7.4 kcal/mol (Table 3). Since a lower energy value indicates a higher probability of binding with target protein, CP and aloin, displaying the lowest docking scores, are preferred ligands for AQP3 and AQP5. It turned out that CP binds to the same protein site as aloin (Figure 2), a component of *Aloe vera* leaf extract that interacts with AQPs and modulates skin hydration activity [24].

**TABLE 3:** Docking scores of affinity of BCP and other substances on AQP3 and AQP5.

№	Substance	Affinity, kcal/mol	
		AQP3	AQP5
1	(−)‐Caryophyllene	−5.40	−6.26
2	Aloin (from *Aloe vera* leaf extract)	−6.56	−7.16
3	Trimethylglycine	−2.92	−3.10
4	Glyceryl glucoside	−4.98	−5.20

**FIGURE 3:**
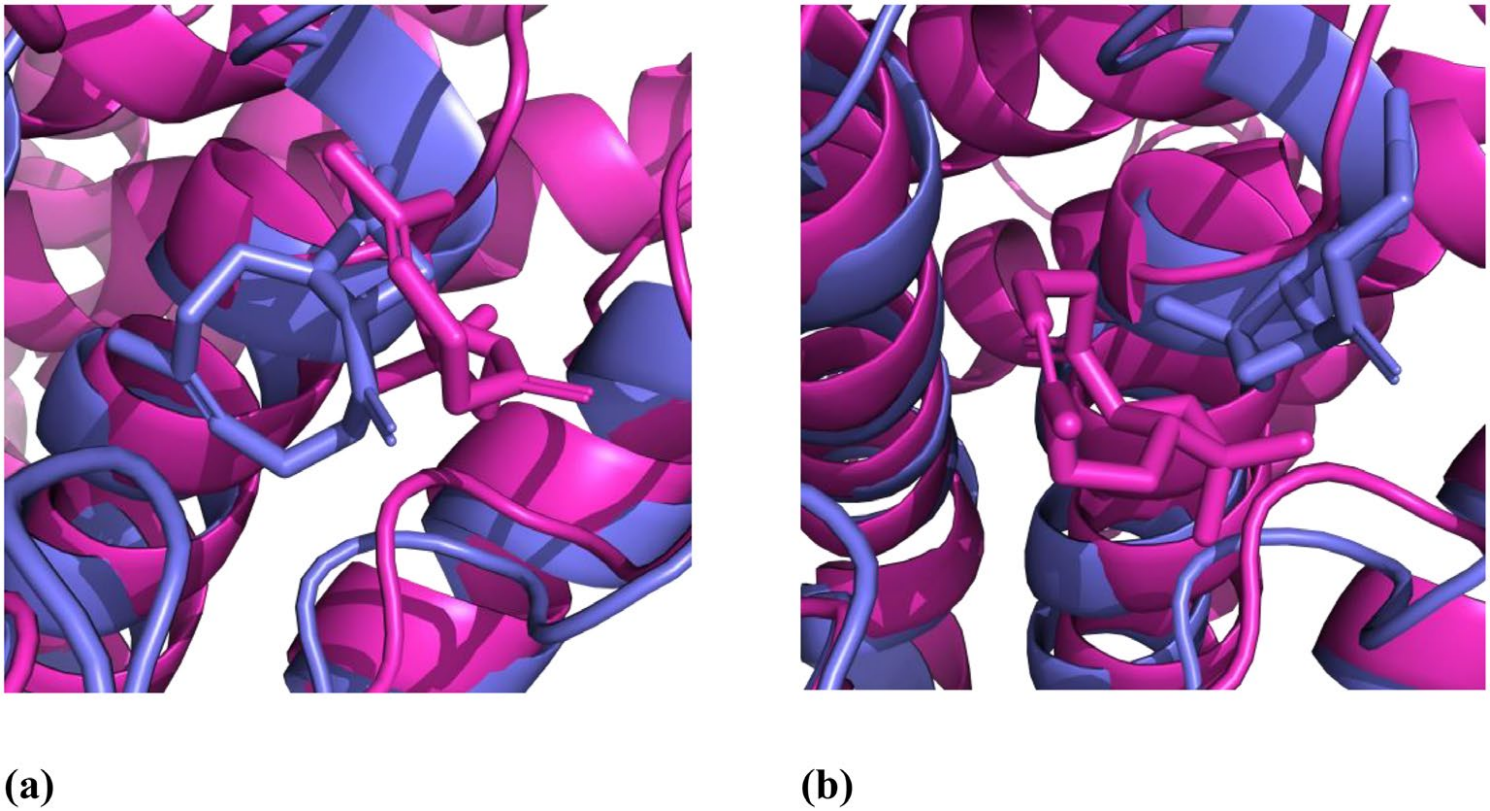
Comparing positions of the CP after MD: (a) AQP3; (b) AQP5. Purple color represents docking positions of CP and proteins; magenta represents position of CP and proteins stable positions after MD.

Molecular docking provides only an initial estimate of binding affinity and is inherently limited by the inaccuracies of scoring functions in reliably predicting true binding free energies [61]. For further validation docking position of (−)‐Caryophyllene (Diffdock‐confidence score equal 0.32 for AQP3 and 0.24 for AQP5), MM/PBSA analysis was used based on the Poisson–Boltzmann method yielded binding free energies of −4.33 ± 0.61 kcal/mol for AQP3 and −5.12 ±0.86 kcal/mol for AQP5. Analysis of the MD trajectories indicated that (−)‐Caryophyllene remained stably positioned within the corresponding docking binding site throughout the simulation (Figure 3).

### 3.3 In Vitro Evaluation of Cytotoxicity and Keratinocytes Viability

**FIGURE 4:**
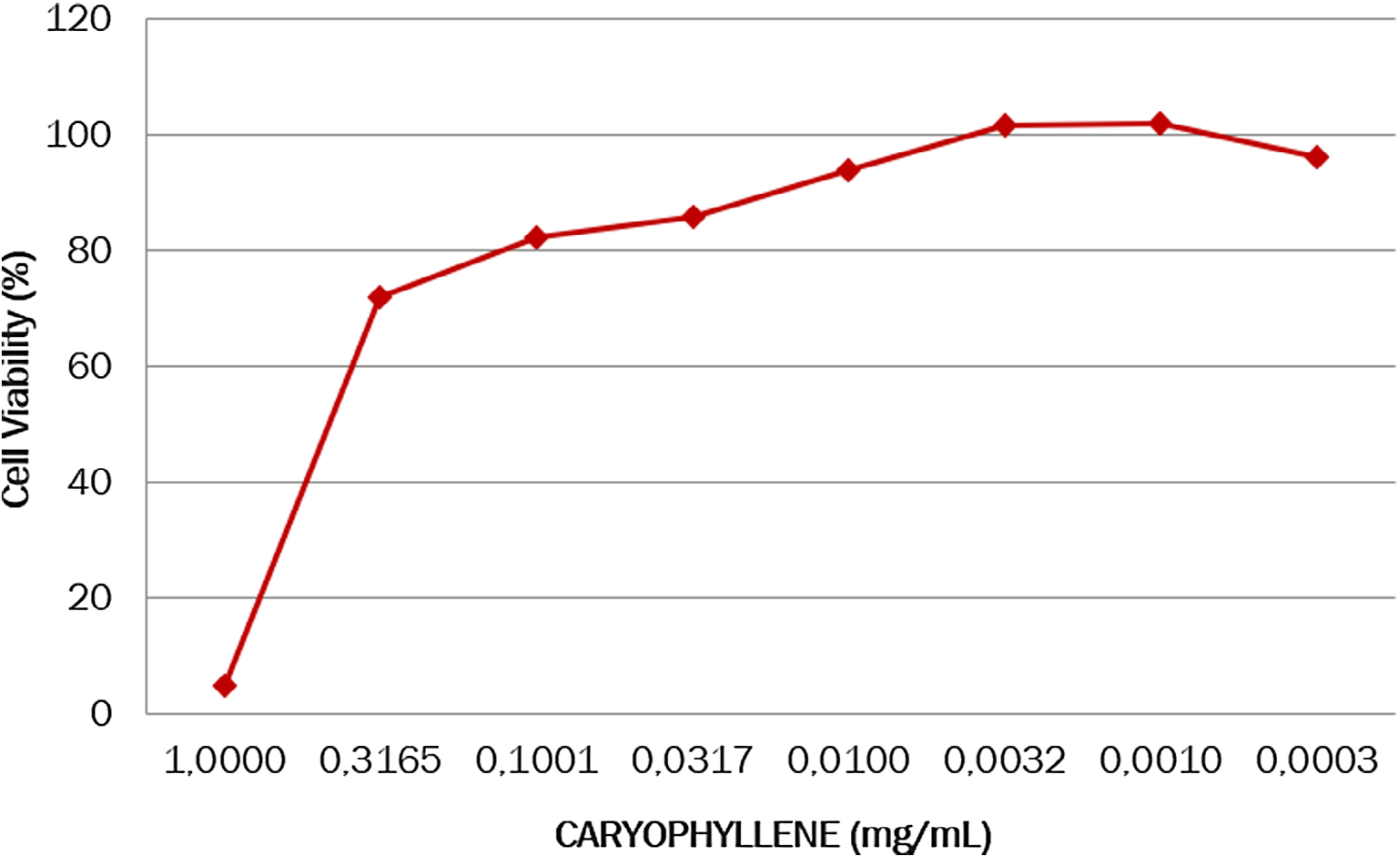
HaCaT keratinocytes viability treated for 48 h with CP.

To evaluate the possibility of CP topical application as a component of pharmaceutics or cosmeceutical formulations targeting AQPs activity, an in vitro skin cell viability assay was performed. The viability of HaCaT keratinocytes after 48 h of incubation with the test compound was determined using the colorimetric MTT method. Human keratinocytes have been chosen as a useful model because of their distribution in both skin epidermis and oral mucosa [62]. According to the experimental results, CP from *Eugenia caryophyllata* was well‐tolerated by cells and did not induce intensive keratinocyte death in the concentration range from 0.01 to 0.30 wt.%. The population of living cells exceeded 70% (EC_70_) after 48‐h incubation with CP at concentrations from 0.0003 mg/mL to 0.3165 mg/mL (Figure 4). The EC_70_ value was estimated to be 0.3165 mg/mL.

The good tolerance of the keratinocyte cell line HaCaT to CP treatment at the tested concentrations of 0.010, 0.0032 and 0.001 mg/mL was confirmed by MTT assay. For a more detailed elucidation of the AQP3 and AQP5 stimulation effect, the tolerated concentrations of the CP with the highest survival rate were selected according to the results of in vitro cytotoxicity assay. The findings indicate CP has high activity even at very low concentrations, potentially allowing the use of small amounts of raw materials in the manufacture of pharmaceutics and cosmeceutical formulations for safety issues and good dermal tolerance.

### 3.4 Quantification of AQPs Amount in Keratinocytes Treated With CP

**FIGURE 5:**
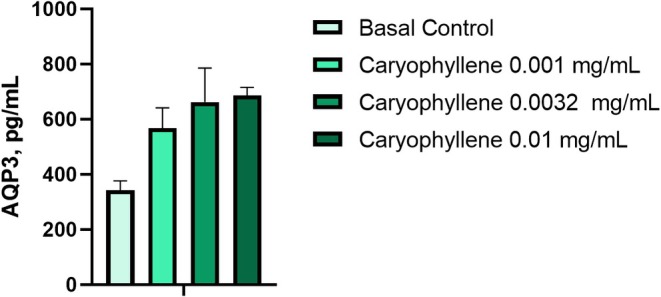
Increased amount of AQP3 in human keratinocytes by the treatment with CP.

To experimentally verify whether BCP, which is predicted to interact with AQPs, an in vitro study using skin keratinocytes was performed. The quantity of AQP3 in HaCaT keratinocytes treated with previously evaluated non‐toxic CP concentrations of 0.001, 0.0032, and 0.010 mg/mL was determined by ELISA. A comparative study was performed where initial cell cultures served as basal control, *Aloe vera* leaf extract standardized for aloin, glyceryl glucoside, and trimethylglycine as positive controls. CP at low tested concentrations significantly overcame the effect of all these constituents known for their ability to increase AQP3 level in a skin keratinocytes model [24] (Figure 5).

Pretreatment keratinocytes with CP for 48 h increased AQP3 amount by 65.46% (*p* < 0.05), 92.87% (*p* < 0.01), and 99.97% (*p* < 0.01) at concentrations of 0.001 (0.03%), 0.0032 (0.08%), and 0.010 (0.25%) mg/mL, respectively, compared to the negative control (Figure 5, Table 4). Previously, it was revealed that the novel combination of *Aloe barbadensis* leaf extract and trimethylglycine in concentrations of 0.01 and 1.00 wt.% respectively increased the amount of AQP3 up to 118.82% [24]. The stimulatory effect observed after treatment with CP in lower concentrations on AQP3 amounts indicates its potential ability to improve the water flow activity and provide intensive skin hydration further.

**TABLE 4:** Modulation of AQP3 amount in human keratinocytes by the treatment with CP in various concentrations.

Tested sample	Concentration, mg/mL	AQP3, pg/mL	Increase in AQP3 level compared to the basal control, %
Basal control	—	343.14 ± 33.27	—
CP	0.001	567.74 ± 73.86	+65.46*
	0.0032	661.79 ± 123.37	+92.87**
	0.01	686.17 ± 29.14	+99.97**

*Note:* Significance levels according to statistical analysis (ANOVA, Bonferroni): *p < 0.05; **p < 0.01.

Altered expression of AQP3 is partially correlated with severity of skin xerosis, xerostomia and Sjögren's syndrome, therefore CP could be considered as a novel phytochemical modulating AQPs amount and normalizing hydration in skin epidermal cells especially [63, 64]. Higher levels of AQP3 can be associated with intensive skin hydration, deep and water transport, and therefore, prophylaxis of skin dryness and skin xerosis. Moreover, CP could enhance wound healing due to known anti‐inflammatory effects through multiple mechanisms related to IL‐1ß and TNF‐α levels in oral mucosa and skin [65, 66].

**FIGURE 6:**
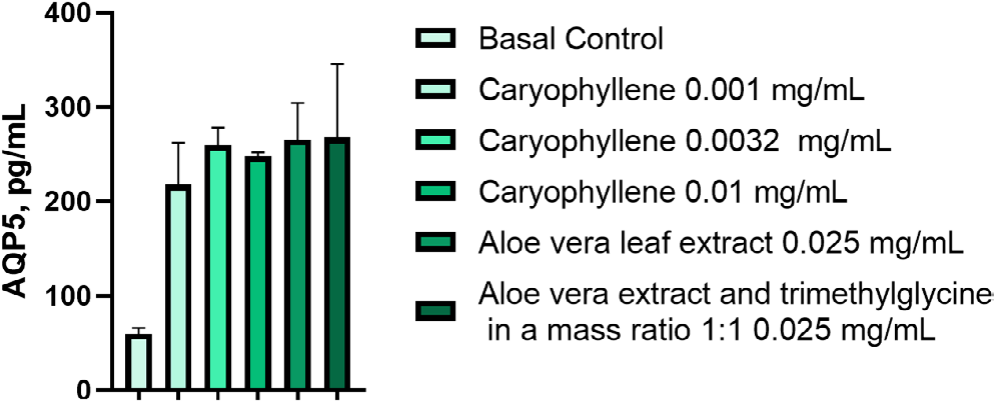
Increased amount of AQP5 in human keratinocytes by the treatment with CP.

Determination of the AQP5 quantity in keratinocytes treated with compounds was performed as described above in the same approach as AQP3 determination. CP was found to significantly increase AQP5 amount by 264.89% (*p* < 0.05), 335.77% (*p* < 0.01), and 332.26% (*p* < 0.01) at concentrations of 0.001, 0.0032, and 0.010 mg/mL, respectively, compared to the basal control. A comparable effect to CP of 0.0032 mg/mL on AQP5 level was obtained by applying *Aloe vera* leaf extract alone and in combination with trimethylglycine in a ratio of 1:1 at a significantly higher concentration of 0.25 mg/mL (Figure 6, Table 5). These findings highlighted a high activity of CP substance and modulation of AQP targets in comparison with well‐known cosmetical raw materials.

**TABLE 5:** Modulation of AQP5 amount in human keratinocytes by the treatment with CP in various concentrations.

Tested sample	Concentration, mg/mL	AQP5, pg/mL	Increase in AQP5 level compared to the basal control, %
Basal control	—	59.58 ± 6.27	—
CP	0.001	217.41 ± 44.83	+264.89*
	0.0032	259.64 ± 18.66	+335.77**
	0.01	257.55 ± 4.41	+332.26**
*Aloe vera* leaf extract	0.025	265.28 ± 38.94	+345.24**
*Aloe vera* extract and trimethylglycine in a mass ratio 1:1	0.025	267.44 ± 77.92	+348.86**

*Note:* Significance levels according to statistical analysis (ANOVA, Bonferroni): *p < 0.05; **p < 0.01.

Considering that AQP5 is a key water channel like other exocrine glands in the salivary gland tissues, it is possible to hypothesize that CP could possess preventive effects on xerostomia, promote salivary secretion and oral cavity hydration based on the evaluated conditions. Thus, CP has a potent action in AQPs modulation compared to the control substances. CP applied in small concentrations from 0.0032 to 0.01 mg/mL significantly increased production of both AQP3 and AQP5 in HaCaT keratinocytes formed skin epidermis and oral mucosa layers. To achieve a similar effect on AQPs expression level, several times higher concentrations of *Aloe vera* extract, the combination of *Aloe vera* extract with trimethylglycine, glyceryl glucoside or trimethylglycine alone are required.

Thus, CP has a similar effect on AQP5, increasing water transport and hydration effect. Lack of expression or decreased transport activity of AQP5 has been associated with severe pathologies such as xerostomia and Sjogren's syndrome [18, 19]. In addition, the AQP5 amount may decrease during inflammation caused by bacterial lipopolysaccharide, leading to dryness and pain [17]. Based on in vitro findings, it is plausible to hypothesize that CP application could help alleviate some dermal and oral pathologies, but this hypothesis requires full validation and massive proof‐of‐concept in appropriate ex vivo models of skin and salivary gland function and extended clinical trials.

Considering the results of *in silico* assay, the high activity of CP may be a result of its good binding of CP to AQPs cavity. In this case, CP has a dual effect on epidermal cells. On the one hand, binding may increase the opening time of AQPs channels, leading to increased water and glycerol transport into epidermal cells in tissues. On the other hand, the observed increase in AQP protein levels could hypothetically involve other epigenetic mechanisms as suggested for other AQP regulators, e.g., histone deacetylase inhibition, but not limited [8].

### 3.5 Chemical Analysis of Cosmeceutical Formulations With CP

Under GC–MS experimental conditions, 0.477% of CP was discovered in the cosmeceutical formulation sample (Table 6). CP oxide and its isomers were detected and estimated in the shower gel, since it in high concentration is suspected to cause allergic contact dermatitis. Through GC–MS, total content of CP oxide and its isomers was estimated as 0.011% in the shower gel sample, providing safe effect on skin during topical application.

**TABLE 6:** The constituents of shower gel sample with CP identified by GC–MS.

№	Compound	RT, min	*m/z*	Content, g/kg	Relative content, %
1	α‐Cubebene	16.119	105.00	0.03	0.003
2	α‐Copaene	16.586	105.00	0.20	0.020
3	trans‐Caryophyllene	17.095	93.00	0.01	0.001
4	β‐Caryophyllene	17.353	133.00	4.77	0.477
5	α‐Humulene (α‐ Caryophyllene)	17.800	93.00	0.84	0.084
6	trans‐Caryophyllene oxide	19.160	79.00	0.11	0.011
7	(−)‐Isocaryophyllene oxide	19.553	79.00		
8	(−)‐Caryophyllene oxide	19.601	79.00		

### 3.6 Quantification of AQP3 Amount in Keratinocytes Treated With the Formulation With CP

**FIGURE 7:**
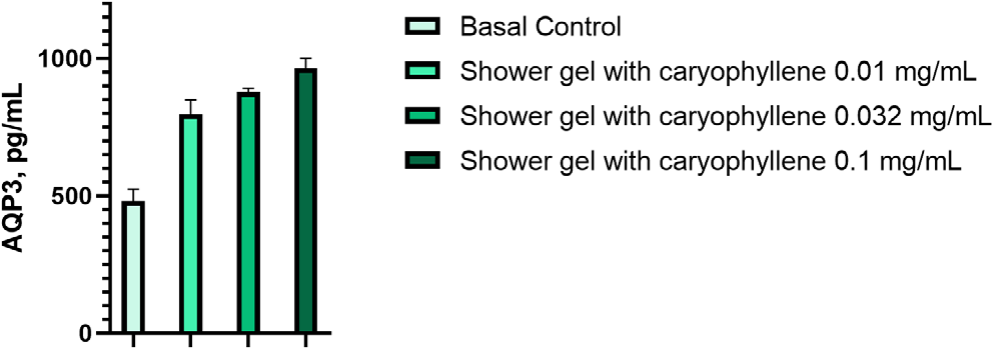
Increased amount of AQP3 in human keratinocytes by the treatment with the CP shower gel formulation.

Figure 7 shows the stimulation effect of the evaluated product on AQP3 levels in HaCaT keratinocytes cultures. Previous treatment of cell culture with the shower gel with CP at concentration of 0.1 wt.% (Table 1) was able to increase AQP3 amount by 100.15%, 82.32% and 65,34% at evaluated concentrations of 0.10, 0.032 and 0.01 mg/mL, in comparison to the basal control (*p* < 0.001).

The results obtained for the shower gel allow us to infer that the CP from *Eugenia caryophyllata* is capable of promoting an increase in AQP3 levels in skin keratinocytes in vitro. Even applying the rinse‐off product, it is possible to maintain skin health for a long‐term period highlighting the promising role in improving skin hydration, prophylaxis of skin dryness, and skin xerosis. Improvement of water transport across cell membranes could lead to intensive skin hydration, skin barrier recovery, and pH‐control [24], therefore AQP3 as a target could be actual for research of novel phytochemicals from essential oils and plants based on them. Meanwhile, long‐term exposure of the cosmetic formulation with CP on skin cells for 48 h limits us to claim an instant AQP3 modulation and consequently skin hydration properties needed for further research and validation.

### 3.7 Dermatological Evaluation of Topical Formulations With CP

Based on the results of in vitro activity, CP can be considered as an active ingredient of cosmeceutical formulations and pharmaceutics for topical application targeting AQP3 and AQP5. To assess the tolerability and efficacy of the phytoconstituent, two topical formulations of shower gels containing 0.03% and 0.10% CP were developed in laboratory conditions.

For dermatological tolerance assessment, shower gel bases supplemented with 0.03 wt.% or 0.10 wt.% CP were applied to the intact skin of the outer arm of 12 adult subjects enrolled in the study. After a single application, the products were kept in contact with the skin for 48 h under a semi‐occlusive patch. Dermatological evaluation after 15 min, 1 h, and 24 h of patch removal revealed no signs of irritation or sensitization regardless of the age and sex of volunteers. The irritation index for both concentrations was evaluated as 0 for all clinical research participants, concluding absence of irritating potential. No one had any skin reactions, erythema, or oedema either. CP at the concentrations of 0.03 wt.% and 0.10 wt.% was well‐tolerated by all participants during the clinical research. Therefore, according to the evaluation scale, shower gels with CP 0.03 wt.% and 0.10 wt.% can be classified as not skin irritating.

**FIGURE 8:**
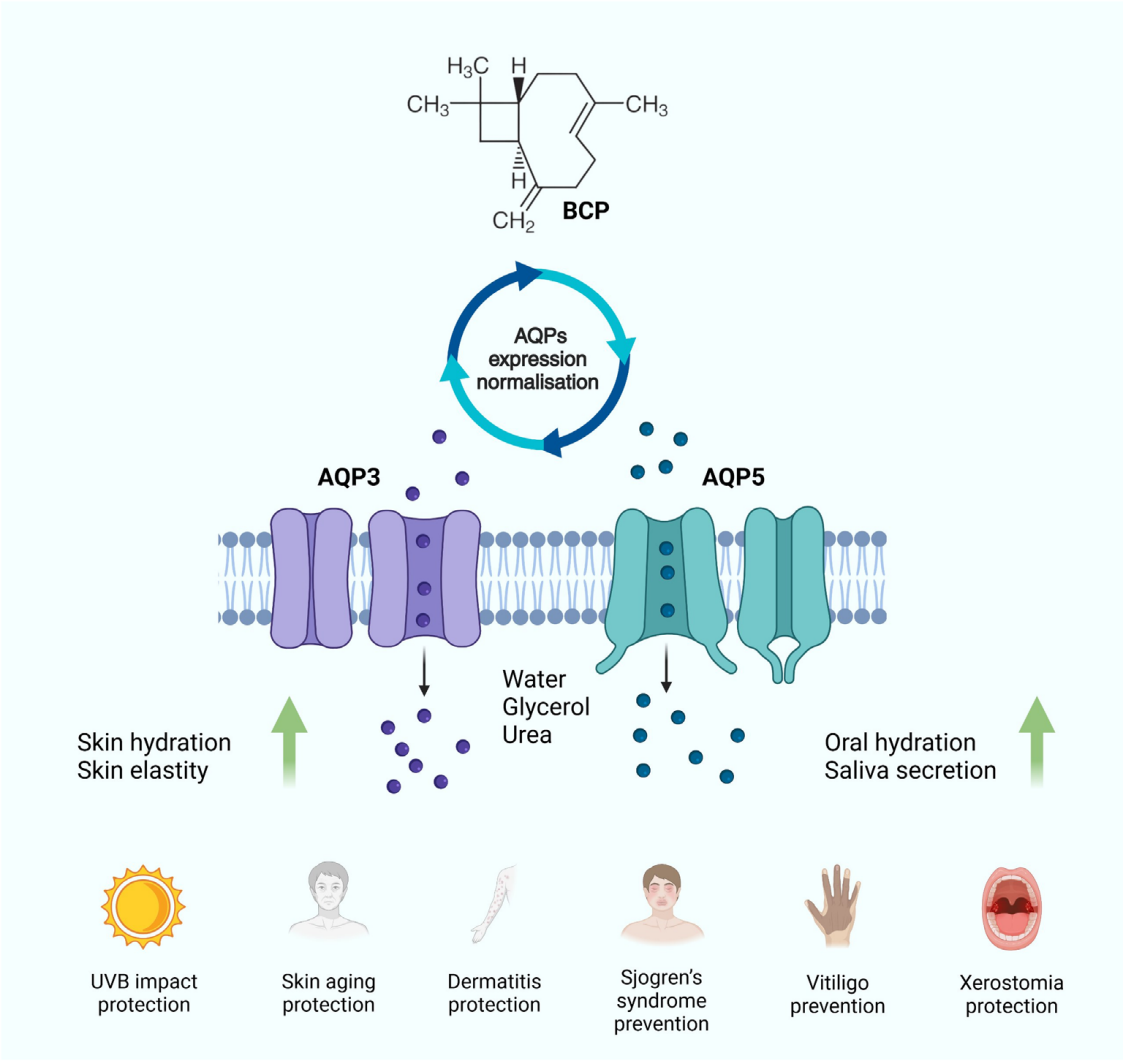
CP effects on skin and oral health through AQPs modulation.

Thus, CP can be considered as a promising active substance of plant origin for both cosmeceutical formulations and pharmaceutics. Since CP at low concentrations effectively increases the amount of AQPs and modulates their activity, likely by binding with main cavities of channels and providing deep transepidermal water flux in all 5 layers of the epidermis, this therapeutical approach may be used to reduce the incidence and severity of skin conditions such as xerosis, atopic dermatitis in infants, children and adults, psoriasis, eczema, contact dermatitis, and hyperkeratosis. It is also conceivable that CP treatment is likely to be helpful in stimulation of saliva secretion and oral mucosa hydration by affecting the level of AQP5 in salivary gland tissues (Figure 8).

## 4 Conclusion

*Eugenia caryophyllata* is enriched by various terpenes and terpenoids with promising therapeutical potential, including caryophyllene (CP). CP and its isomers consist of 93.219% of *Eugenia caryophyllata* essential oil. At the same time, CP oxide isomers having high allergic potential have a content of < 1%, confirming non‐skin irritating activity. CP has a good toxicological profile in a concentration of 0.001–0.3 mg/mL on HaCaT keratinocytes in vitro. CP at tested concentrations ranging from 0.003 to 0.01 mg/mL significantly increased the amount of AQP3 and AQP5 in skin keratinocytes, showing higher activity compared to *Aloe vera* leaf extract, trimethylglycine, their combination in a mass ratio of 1:1 and glyceryl glucoside. These effects suggest a promising beneficial role in skin and oral hydration process through increased AQP amount and probably accelerated water transport across membranes. These findings underscore the therapeutical role of the CP as a phytochemical for the development of cosmeceuticals and pharmaceutics aimed at prophylaxis and treatment of dryness‐related conditions, such as skin xerosis and xerostomia. Further extended proof‐of‐concept, including toxicological evaluation, dermatological tolerance and biological pathways on skin and salivary gland ex vivo models, placebo‐controlled clinical research and standardization of CP in the final substance for quality control are needed.

## Author Contributions

V.F.: conceptualization, data curation, formal analysis, investigation, methodology, project administration, resources, validation, writing – original draft, writing – review and editing. A.Y.: conceptualization, data curation, formal analysis, investigation, methodology, project administration, resources, validation, writing – original draft, writing – review and editing. E.I.: formal analysis, investigation, methodology, resources, software, validation, visualization, writing – original draft, E.P.: formal analysis, investigation, visualization, writing – original draft. B.F.C.: data curation, investigation, methodology, project administration, resources, validation, writing – original draft. S.E.: data curation, investigation, methodology, project administration, resources, validation, writing – original draft.

## Disclosure

Generative AI Statement: The authors declare that no Generative AI was used in the creation of this manuscript.

## Ethics Statement

The semi‐occlusive patch test on healthy volunteers was approved by the International Ethics Committee of CE.way Regulatory Consultants Ltd. (protocol 2023–0829 dated December 13th, 2023). The clinical research was strictly conducted in accordance with the local legislation and institutional requirements.

## Conflicts of Interest

Authors V.F., E.P., and A.Y. were employed by SkyLab A.G. The remaining authors declare that the research was conducted in the absence of any commercial or financial relationships that could be construed as a potential conflicts of interest. The author(s) declared that they weren't editorial board members of Wiley at the time of submission. This had no impact on the peer review process and the final decision.

## Data Availability

The data that support the findings of this study are available from the corresponding author upon reasonable request.
